# Dr. Rigbys’ Obstetric Memoranda

**Published:** 1872-06

**Authors:** 


*Dr. Rigby's Obstetric Memoranda.* Fourth Edition revised and enlarged. By ALFRED MEADOWS, M. D. etc. Philadelphia: Lindsay & Blakiston 1872.

